# Circ-CUL2/microRNA-888-5p/RB1CC1 axis participates in cisplatin resistance in NSCLC via repressing cell advancement

**DOI:** 10.1080/21655979.2021.2024395

**Published:** 2022-01-22

**Authors:** HengQi Chen, Fang Li, Qi Xue

**Affiliations:** Deparment of Thoracic Surgery, Cancer Hospital Chinese Academy of Medical Sciences, Beijing, China

**Keywords:** Circular RNA, Circular CUL2, microRNA-888-5p, RB1CC1, Non-small cell lung cancer

## Abstract

Elevated evidences manifest that circular RNAs (circRNAs) are vital in human tumor advancement and chemotherapy resistance. The study was to explore the character of Circ-CUL2 in non-small cell lung cancer (NSCLC). Firstly, the expression of circ-CUL2, microRNA (miR)-888-5p and RB1CC1 was detected in human NSCLC tissues and cell lines by reverse transcription quantitative polymerase chain reaction or Western blot. Then, cell counting kit (CCK)-8, plate clone, Transwell assays, and flow cytometry were applied to separately detect the impacts of circ-CUL2 on proliferation, migration, invasion, apoptosis and cisplatin (DDP) resistance of A549/DDP cells. In this study, exploration of the biological function of Circ-CUL2 was via the Circ-CUL2/miR-888-5p/RB1CC1 axis. The results manifested circ-CUL2 and RB1CC1 were down-regulated in NSCLC tissues and cell lines, while miR-888-5p was up-regulated. Elevated Circ-CUL2 or refrained miR-888-5p repressed A549/DDP cell progression with depressive DDP resistance. Circ-CUL2 curbed miR-888-5p, which targeted RB1CC1. Restrained RB1CC1 turned around the impacts of Circ-CUL2 on the cells. All in all, Circ-CUL2 is anti-NSCLC via miR-888-5p/RB1CC1 axis, enhancing the sensitivity of A549/DDP cells to DDP. Hence, Circ-CUL2 is supposed to be a novel biomarker offering a brand-new strategy for NSCLC therapy.

## Graphical abstract

**Figure:**
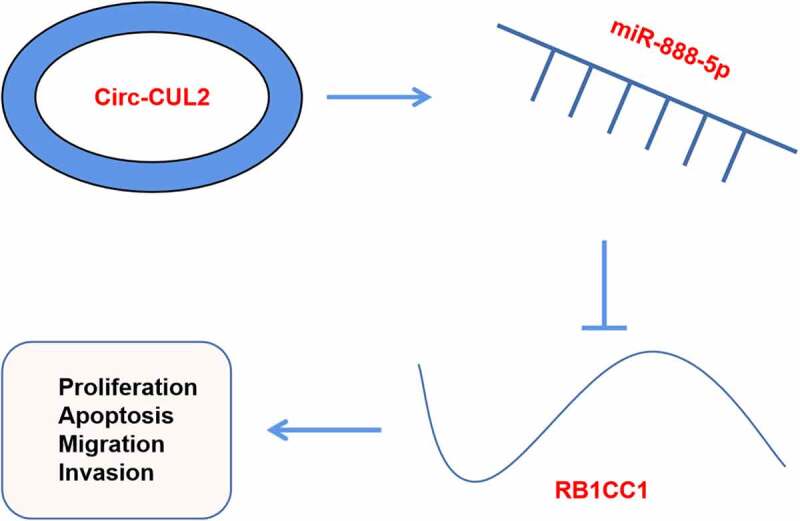

## 1 Introduction

Lung cancer (LC) is a momentous cancer with surprising morbidity and mortality, and listed as a main reason of cancer-linked deaths worldwide [1,2]. It is reported the link of LC’s incidence with ongoing lung transfection, smoking, exposure to carcinogens, and susceptibility induced via gene mutations [3–5]. Nevertheless, the molecular mechanism of LC is still far from completely explicit. Non-small cell LC (NSCLC)’s histological and cytological features are apparently disparate from small cell LC, and epidemiological reports clarify that non-small cell LC takes up over 80% of LC cases worldwide [6]. A great deal of chemotherapy drugs, like cisplatin (DDP) and Vinorelbine, are extensively applicated in NSCLC therapy, but frequent drug resistance usually results in adverse reactions and elevated recurrence rates, seriously influencing their efficacy [7,8]. Uncovering the molecular mechanism of chemotherapeutic drug resistance is vital for overcoming chemotherapeutic drug resistance and new drugs exploitation.

Circular RNAs (circRNAs), a group of non-coding RNA molecules, modulate functional genes via sponge-binding miRNA and interacting with RNA-binding proteins [9,10]. Recent reports have established the crucial modulators of circRNAs in all kinds of biological processes and pathogenic conditions, like the presence of NSCLC [11,12]. For instance, CircRNA_101237 is memorably elevated in both tissues and cell lines of NSCLC, and supposed to function as a brand-new oncogene in NSCLC advancement via targeting miR-490-3p [13]. What’s more, circRNA is also apparently implicated with the multi-resistance of LC cells to chemotherapy drugs [14,15]. The character of circRNA CUL2 as a brand-new RNA for DDP chemotherapy resistance in NSCLC cells has not been clarified.

MicroRNAs (miRNAs), diminutive non-coding RNAs, modulate their expression via targeting specific 3ʹuntranslated region of mRNA. MiRNAs are linked with all kinds of physiological and pathological processes of cancer [16], and available to control disparateiation, infiltration, radiation resistance and chemotherapy resistance of cancer cells [17]. A study has proposed elevation of miR-888-5p in hepatocellular carcinoma and facilitation of metastasis of cancer cells [18].

RB1CC1, also named as FIP200 (200kda FAK family interacting protein), is a tumor suppressant [19,20]. A recent study has manifested the repression of RB1CC1 in renal cell carcinoma (RCC) cells advancement [21]. The study was to clarify the relative Circ-CUL2 in NSCLC tissues and cells, its impacts on the biological behavior of A549/DDP cells, and the potential character in DDP resistance. The results affirmed that Circ-CUL2 repressed A549/DDP cell advancement, and enhanced DDP sensitivity of A549/DDP cells via miR-888-5p/RB1CC1 axis. Hence, Circ-CUL2 is supposed to be a biomarker and therapeutic target for NSCLC.

## 2 Materials and methods

### 2.1 Patients and specimens

Gain of 46 pairs NSCLC and the paired adjacent anticancer tissues was from NSCLC patients undergoing surgery in Cancer Hospital Chinese Academy of Medical Sciences. No reception of chemotherapy or radiotherapy before surgery was in all patients with confirming pathological examination as NSCLC. Store of all tissue samples was in liquid nitrogen for RNA extraction. All patients were followed up for 60 months.

### 2.2 Cell Culture

Culture of human NSCLC cell lines (A549, NCI-H1299, PC-9 and LLC) and normal human liver cell line (BEAS-2B) (China Type Culture Collection Center, Wuhan, China) was in Roswell Park Memorial Institute-1640 medium (Gibco) consisting of 10% fetal bovine serum (FBS) (PAN, Bavaria, Germany).

### 2.3 Drug-resistant cell model

Seeding of the logarithmic A549 cells was in petri dishes at a density of 5 × 10^5^ cells/mL. Introduction of the cells was with 1 mg/L DDP (Jiangsu Hengrui Pharmaceutical Co., Ltd.). After 3 times of passages, stable growth of the cells was in the medium consisting of drugs. During this time, elevation of DDP concentration was to 2 mg/L and gradual augment was done until the cells clarified steady growth at 10 mg/L. Name of the established cell line was A549/DDP cells.

### 2.4 Cell transfection

Synthesis of oe/sh-CUL2, miR-888-5p inhibitor/mimic, si-RB1CC1 and their negative control (NC) lentivirus vectors was via GenePharma (Shanghai, China). In line with the manufacturer’s instructions, introduction of lentiviruses was into cells via polypropylene (Shanghai Hanssen Biotechnology Co., Ltd., China). Removing the un-transfected cells was with puromycin (2 µg/mL).

### 2.5 Cell counting kit (CCK)-8

Seeding of A549/DDP cells was into 96-well plates. Culture of the cells was with disparate doses of DDP and then addition was with 10 mg/mL CCK-8 reagent (Beyotime, Shanghai). Determination of cell viability was in line with manufacturer’s instructions. Measurement of the absorbance at 450 nm was with a microplate reader, and determination of the half maximal inhibitory concentration (IC50) of DDP in A549/DDP cells was conducted.

### 2.6 Plate cloning

Seeding of A549/DDP cells in the logarithmic phase was into 6-well plates at a density of 600 cells per well. Culture of the 6-well plates was clarified until a cell clone was seen to the unaided eye in a petri dish. Fixation was with 10% formaldehyde and stain was with crystal violet (Beyotime, Shanghai), and count of clones with over 50 cells was under a low power microscope.

### 2.7 Transwell

Determination of cell migration and invasion capacities was via Transwell chambers (pore size 8.0 mTM; EMD Millipol, Billerica, Ma, USA) and Matrigel (1: 9) (Corning, USA). Suspension of the transfected A549/DDP cells (4 × 10^5^ cells) was in 200 µL serum-free Dulbecco’s Modified Eagle Medium, and addition of the upper chamber was with or without 10 μL matterige, and the lower chamber was full of 500 μL complete medium consisting of 10% FBS. After incubation, removing of the cells remaining on the upper surface of the membrane was done. Fixation of the basal cells was with 4% paraformaldehyde, and stain was with 0.1% crystal violet (Beyotime, Shanghai), and count via photographing was under an upright microscope (Nikon, Japan).

### 2.8 Flow cytometry

Harvest of A549/DDP cells in logarithmic growth period was to prepare single-cell suspension and seeding was in 25 cm^2^ culture flask. The old culture medium was abandoned behind overnight adherence. Culture of the experimental group was with 0.3% FBS culture medium, and that of the control was with equal volume phosphate buffer saline culture medium. Behind harvest of the cell supernatant, detachment of the cells was with trypsin without ethylene diamine tetraacetic acid with collection. The rest steps were followed in line with the instructions of AnnexinV-propidium iodide apoptosis kit. Detection of the cell apoptosis was via flow cytometry.

### 2.9 Quantitative Real-Time PCR (RT-qPCR)

Extraction of total RNA was from tissues or cells via TRIzol reagent (Invitgen, USA) with synthesis of complementary DNA via CircRNA reverse transcription kit (Guangzhou GYSai Biotechnology Co., Ltd.). Conduction of RT-qPCR was via CircRNA real-time PCR detection kit on ABI7500 real-time PCR system (Applied Biosystems, Foster City, California, USA). Detection of Circ-CUL2 was with glyceraldehyde-3-phosphate dehydrogenase (GAPDH) as a loading control and calculation of the relative circRNA was via 2^ΔΔCt^ method. Design and synthesis of all the primers were via Guangzhou RiboBio, as clarified in Table 1.

**Table 1.** RT-qPCR primers

Genes	Primer sequences (5ʹ–3ʹ)
CLU2	F: ATGATGAAGACTCTGCTGCTG
	R: CTCCTCCCGGTGCTTTTTG
MiR-888-5p	F: ATGTGGCAGATCCCACAGGAGTTT
	R: ACTGGGTTTGACTTCGTAGCCCTT
RB1CC1	F: TGCTGCACAAGACTCTCACA
	R: CAGCATTTCCTTCTGCTGTG
U6	F: CTCGCTTCGGCAGCACA
	R: AACGCTTCACGAATTTGCGT
GAPDH	F: CGGAGTCAACGGATTTGGTCGTAT
	R: AGCCTTCTCCATGGTGGTGAAGAC

### 2.10 Western blot Analysis

Lysis of the cells was with a lysis buffer consisting of 1 nM phenylmethylsulfonyl fluoride (Beyotime, Shanghai, China) and determination of the protein concentration was via bicinchoninic acid protein analysis kit (Beyotime). Determination of protein separation was via sulfate polyacrylamide gel electrophoresis (Beyotime) and electroblot was onto polyvinylidene fluoride microporous membrane. Incubation was with anti-β-catenin (1: 800), anti-c-Myc (1: 500) and anti-GAPDH (1: 500), and horseradish peroxidase (1: 5000) (all Ruiying, China) coupling with the mouse anti-rabbit Immunoglobulin G secondary antibody. Manifestation of protein bands was with the WestrenBright electrogenerated chemiluminescence kit (Advansta, USA). Finally, application of Image Lab software (Bio-Rad, Hercules, California, USA) was for analysis of the signal strength of the bands.

### 2.11 The luciferase activity assay

Prediction of the binding sites of Circ-CUL2 to miR-888-5p and miR-888-5p to RB1CC1 was via the bioinformatics website (starBase). Verification of the binding of the factors was affirmed. Inserting of corresponding sequences was into pmirGLO (Promega, Madison, Wisconsin, USA) to construct CUL2/RB1CC1-wild and mut types (WT/MUT), which were then co-transfected with miR-888-5p mimic or its NC. Finally, assessment of the luciferase activity was via a dual luciferase reporter gene assay kit (Promega).

### 2.12 Statistical analysis

Results analysis was via GraphPad Software 6.0 (GraphPad Inc., San Diego, CA, USA) and SPSS software (version 24.0, Chicago, IL, USA) (*N* = 3). Presentation of the data was as mean ± standard deviation (SD). Application of student *t* test and one-way analysis of variance (ANOVA) was for comparison of the statistical differences of two or multiple groups, with *χ*^2^ test for evaluation of the association of CirC-CUL2 with clinicopathological features of NSCLC patients. Kaplan–Meier survival analysis and logarithmic rank test were applied to analyze the survival curve. *P* < 0.05 was considered statistically significant.

## 3 Results

### 3.1 Repressive Circ-CUL2 is in NSCLC; the circRNA is implicated with unpleasing prognosis in NSCLC patients

**Figure 1.**
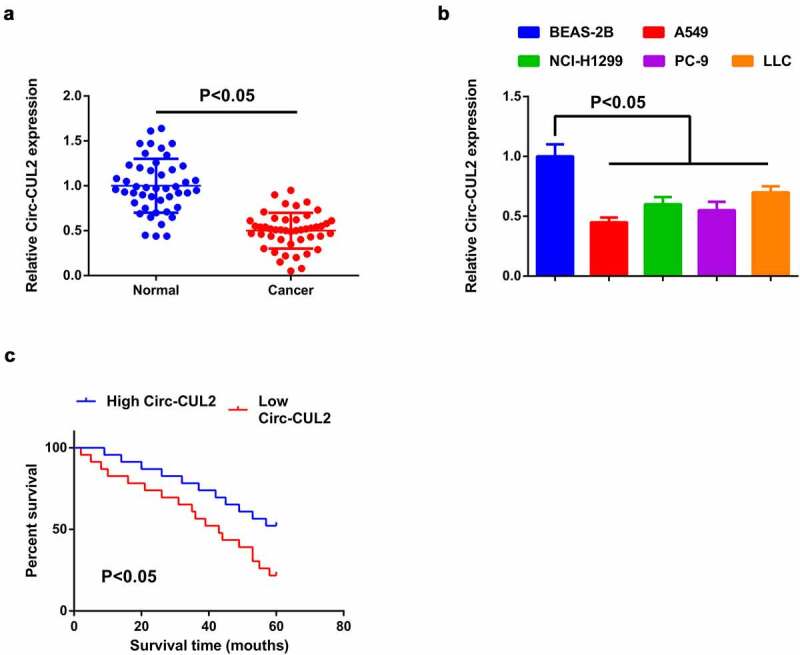
Reduction of Circ-CUL2 is in NSCLC tissues and cells.

For exploration of the character of Circ-CUL2 in NSCLC, detection of Circ-CUL2 in NSCLC and tissues and cells was done, manifesting the decline (Figure 1(a, b)). Meanwhile, A549 cells clarified the most apparent difference, hence, choice of the cells was for subsequent experiments. In view of the dysregulation of Circ-CUL2 in NSCLC tissues and cells, analysis of the association of Circ-CUL2 with the clinicopathological features of NSCLC patients was done. Assignation of the patients was into Circ-CUL2 elevation and repression groups (*n* = 23 separately). Comparison of the clinicopathological parameters of the two was via Pearson Chi-square test. The association of plasma Circ-CUL2 with clinicopathological features was clarified, like age, gender, smoking status, histological classification, tumor size, lymph node metastasis and clinical stage (Table 2). The results manifested that plasma Circ-CUL2 was extremely linked with clinical stage and lymph node metastasis, assuring that CirC-CUL2 was supposed to be a biomarker for NSCLC diagnosis and prognosis. Meanwhile, via Kaplan–Meier survival analysis, it was clarified the elevated overall survival rate of patients with augmented Circ-CUL2 versus that with reduced one (Figure 1(c)), manifesting patients with elevated CirC-CUL2 were more sensitive to drugs, while that with reduced one were more likely to develop drug resistance.

**Table 2.** Association of Circ-CUL2 with clinical features in NSCLC patients

Features	Number	CircCUL2		*P*
		Elevated (*n* = 23)	Reduced (*n* = 23)	
Age				0.531
<60	20	9	11	
60 or more	26	14	12	
Gender				0.758
Male	30	14	16	
Female	16	9	7	
Smoking Status				0.284
Ever	36	16	20	
Never	10	7	3	
Histological classification				0.725
SCC (Squamous cell carcinoma)	18	8	10	
AD (adenocarcinoma or other)	28	15	13	
Tumor Size				0.530
3 or less cm	15	6	9	
>3 cm	31	17	14	
TNM Stage				<0.001
I + II	16	1	15	
III + IV	30	22	8	
Lymphatic metastasis				0.002
Yes	35	13	22	
No	11	10	1

(a and b) RT-qPCR detection of CirC-CUL2 in NSCLC and adjacent tissues (*n* = 46), 4 cell and 1 normal human lung epithelial cell lines. (C) Kaplan–Meier survival analysis to compare the overall survival rates of patients with elevated and reduced circ-CUL2 expression (**Attached**Figure 1).**The effects of circ-CUL2 on the expression of miR-888-5p and RB1CC1.** (A and B) RT-qPCR and Western blot to detect the expression of miR-888-5p and RB1CC1. The data in the figure were measurement data, and the values were manifested as mean ± SD. * vs. the oe-NC, *P* < 0.05; # vs the sh-NC, *P* < 0.05. and vs. the oe-CUL2 + si-NC, *P* < 0.05.

### 3.2 Elevated Circ-CUL2 represses A549/DDP cell advancement with depressive DDP resistance

**Figure 2.**
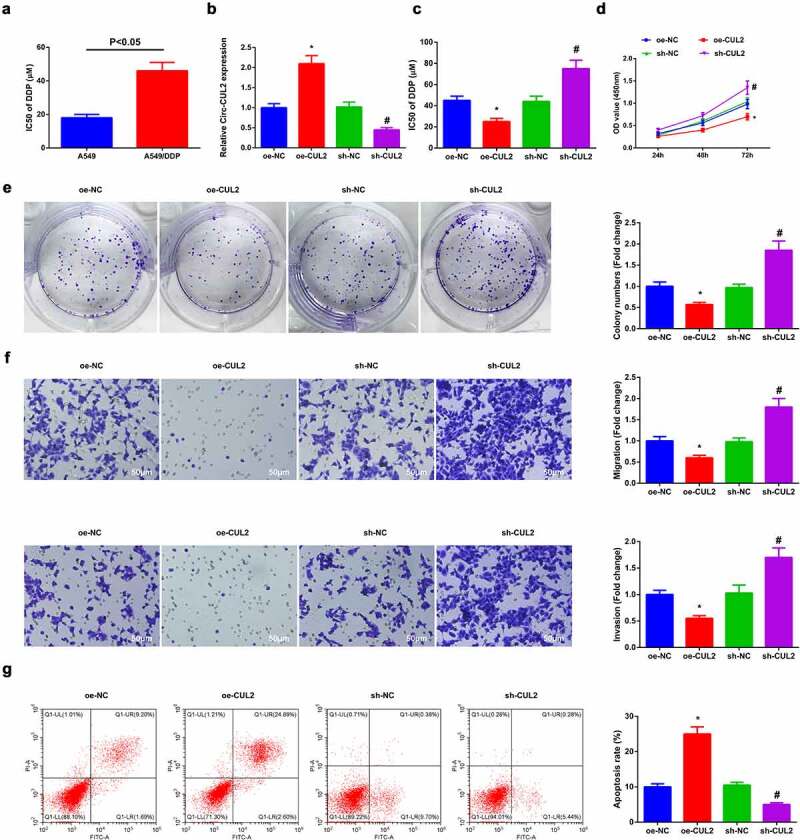
Circ-CUL2 curbs A549/DDP cell advancement.

For exploration of the modulation of Circ-CUL2 on DDP resistance in NSCLC, construction of a DDP resistance cell model was via A549 cells with name as A549/DDP cells. Analysis of IC50 value of DDP in A549 cells and A549/DDP cells was clarified, manifesting the apparent elevation in A549/DDP cells (Figure 2(a)). Then separate transfection of oe/sh-CUL2 and their NC was into A549/DDP cells and the efficiency of lentivirus transfection was confirmed (Figure 2(b)). The results manifested that elevated Circ-CUL2 was available to repress cell advancement with depressive DDP IC50 value, while reduced one was opposite with strengthening DDP resistance (Figure 2(c–g)).

(a) CCK-8 analysis of IC50 value of DDP in A549 and A549/DDP cells. (b) RT-qPCR verification of the successful transfection of Circ-CUL2. (c, d) The influences of Circ-CUL2 on cell proliferation and IC50 value of DDP determined via CCK-8 method. (e) Plate cloning analysis of the impacts of Circ-CUL2 on cell colony. (f) Transwell analysis of the impacts of Circ-CUL2 on cell migration and invasion. (g) The impacts of Circ-CUL2 on apoptosis analyzed via flow cytometry. (B–G) in A549/DDP cells. The data in the figure were all measurement data, and presentation of the values was as mean ± SD; * vs the oe-NC, *P* < 0.05; # vs the sh-NC, *P* < 0.05 (**Attached**Figure 2).**The effects of circ-CUL2/miR-888-5p/RB1CC1 axis on DDP nonresistant cell lines.** (a–b) CCK-8 and colony formation assays to detect cell proliferation. (c) Transwell assay to test cell migration and invasion. (d) Flow cytometry to detect cell apoptosis. The data in the Fig. were measurement data, and the values were manifested as mean ± SD. * vs the oe-NC, *P* < 0.05; # vs the inhibitor-NC, *P* < 0.05. and vs. the oe-CUL2 + si-NC, *P* < 0.05.

Briefly, Circ-CUL2 restrained A549/DDP cell advancement, with enhancing DDP sensitivity.

### 3.3 Circ-CUL2 refrains miR-888-5p

**Figure 3.**
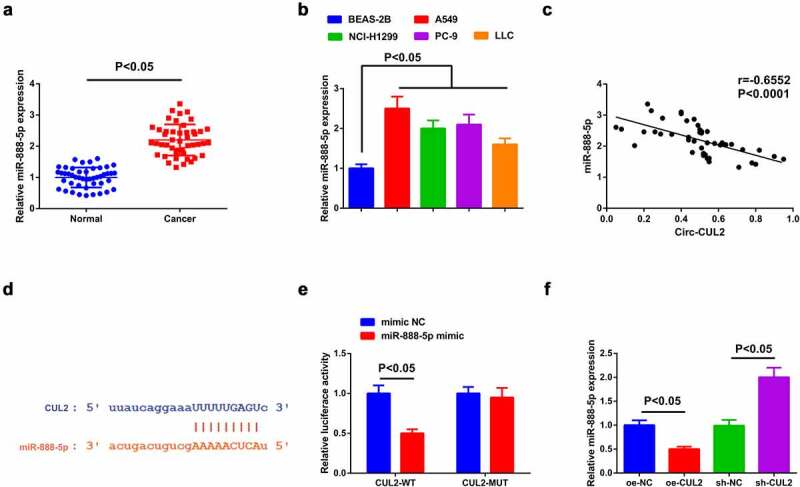
Circ-CUL2 represses miR-888-5p.

Detection of miR-888-5p in NSCLC tissues and cells affirmed the augment (Figure 3(a, b)).

(a, b) RT-qPCR detection of miR-888-5p in NSCLC and adjacent tissues (*n* = 46), cell lines and normal cells. (c) Association of Circ-CUL2 with miR-888-5p. (d) Binding sites of the two. (e) The binding of them verified via the luciferase activity assay. (f) RT-qPCR detection of miR-888-5p in A549/DDP cells in groups. The data in the figure were all measurement data, and presentation of the values was as mean ± SD (*n* = 3), *P* < 0.05.

A speculation was clarified the certain association of Circ-CUL2 and miR-888-5p in NSCLC. To explore whether Circ-CUL2 directly curbed miR-888-5p, application of Pearson test was for analysis of the link of the two in clinical samples, clarifying the negative link of them (Figure 3(c)). Then, via the bioinformatics website was discovered the binding sites of the two (Figure 3(d)). Additionally, reduced luciferase activity was manifested after co-transfection of CUL2-WT and miR-888-5p mimic (Figure 3(e)). Further verification clarified descending miR-888-5p behind augmented Circ-CUL2, which was opposite behind depressing one (Figure 3(f)). Shortly, Circ-CUL2 refrained miR-888-5p. In addition, it was also found that circ-CUL2 promoted the expression of RB1CC1 by repressing the expression of miR-888-5p, and down-regulation of RB1CC1 reversed the acceleration of elevated circ-CUL2 on the expression of RB1CC1 (attached Figure 1(a, b)).

### 3.4 Depressive miR-888-5p refrains A549/DDP cell advancement with constrained DDP resistance

**Figure 4.**
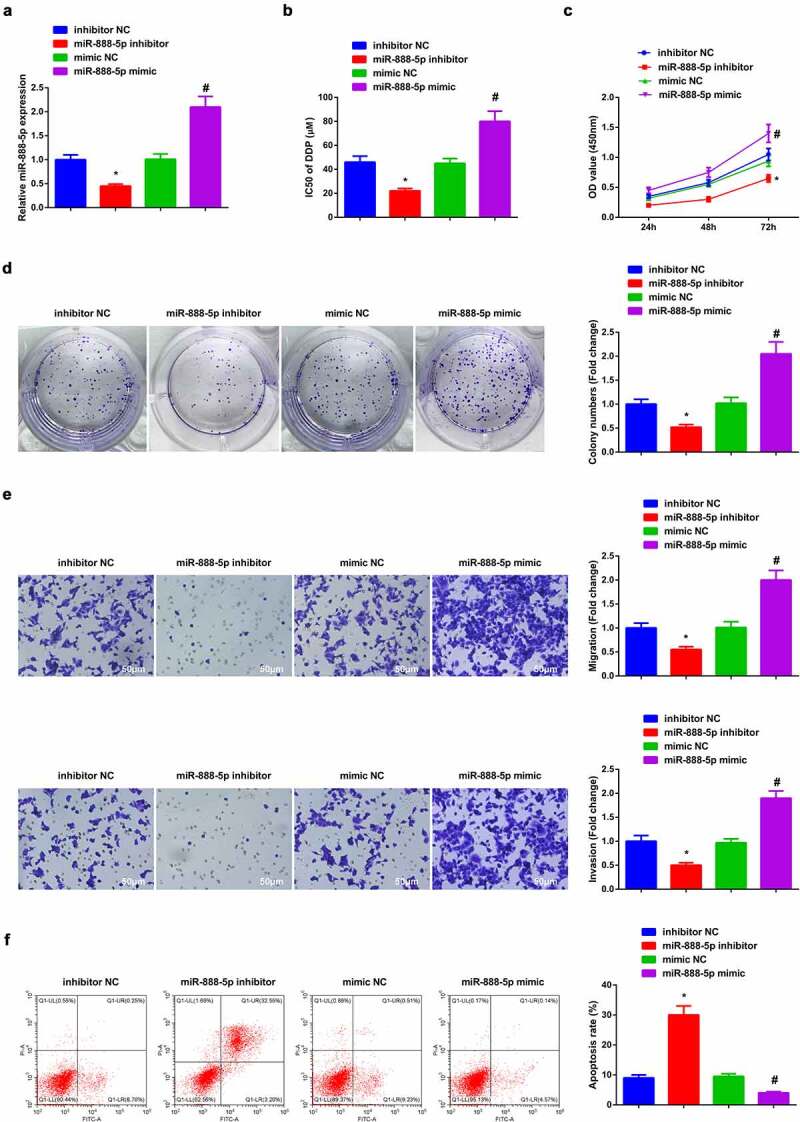
Repressive miR-888-5p refrains A549/DDP cell advancement with descending DDP resistance.

Verification of the successful transfection of miR-888-5p was done (Figure 4(a)). The results clarified that restrained miR-888-5p constrainedA549/DDP cell advancement with depressive IC50 value of DDP, while ascending one was available to enhance DDP resistance with expediting cell progression (Figure 4(b–f)). Briefly, refrained miR-888-5p curbed A549/DDP cell advancement with constrained DDP resistance.

(a) RT-qPCR verification of the successful transfection of miR-888-5p in A549/DDP cells. (b, c) CCK-8 method’s determination of the impacts of miR-888-5p on A549/DDP cell proliferation and IC50 value of DDP. (d) Plate cloning analysis of the impacts of miR-888-5p on cell colony. (e) Transwell analysis of the impacts of miR-888-5p on cell migration and invasion. (f) The impacts of miR-888-5p on apoptosis analyzed via flow cytometry. The data in the figure were all measurement data, and presentation of the values was as mean ± SD; * vs the inhibitor NC, *P* < 0.05; # vs the mimic NC, *P* < 0.05.

### 3.5 MiR-888-5p targets RB1CC1

**Figure 5.**
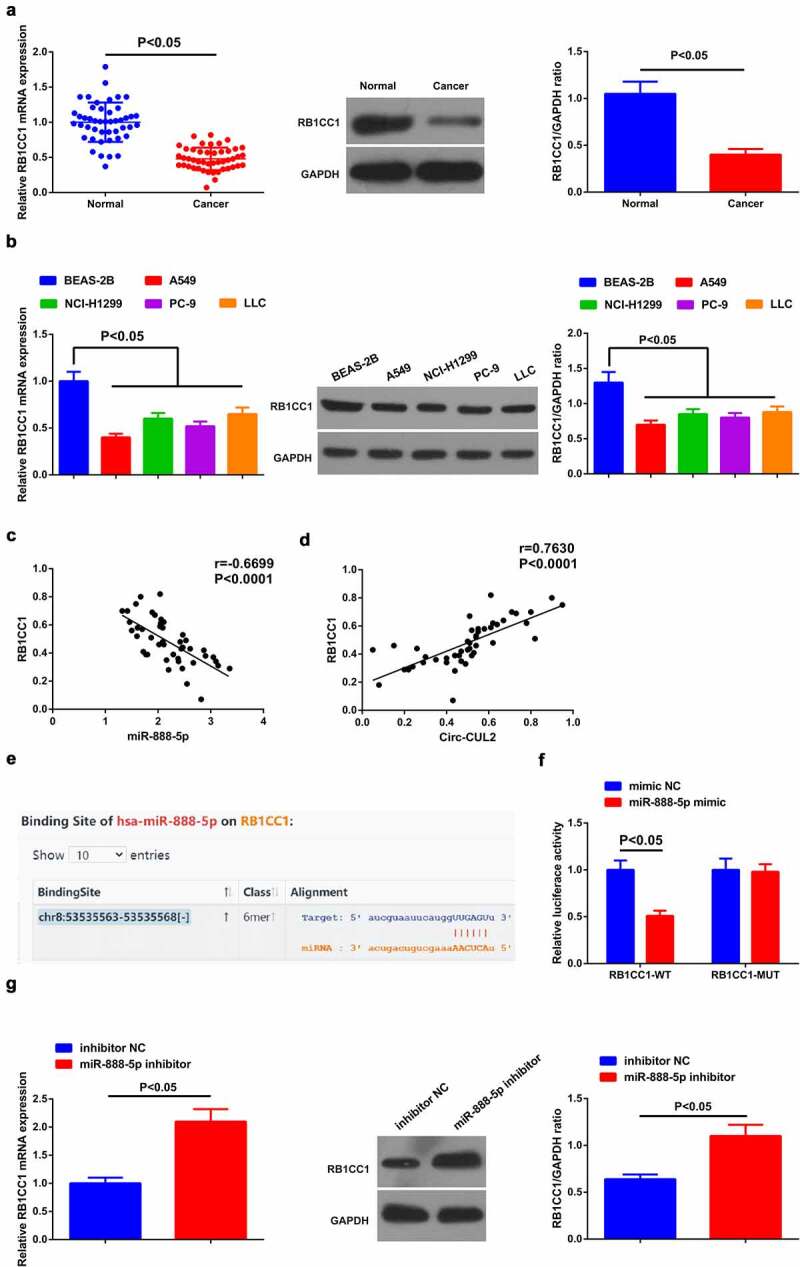
MiR-888-5p targets RB1CC1.

Detection of RB1CC1 in NSCLC tissues and cells clarified the decline (Figure 5(a, b)). Meanwhile, a negative correlation of miR-888-5p with RB1CC1 was observed in clinical samples (Figure 5(c)), while positive link was clarified in Circ-CUL2 and RB1CC1 (Figure 5(d)).

(a, b) RT-qPCR and Western blot detection of RB1CC1 in NSCLC and adjacent tissues (*n* = 46), cell lines and normal cells. (c) The link of miR-888-5p and RB1CC1. (d) The link of Circ-CUL2 with RB1CC1. (e) Target sites of miR-888-5p and RB1CC1. (f) The luciferase activity assay verification of the targeting of the two. (g) RT-qPCR and Western blot detection of RB1CC1 in A549/DDP cells of groups. The data in the figure were all measurement data, and presentation of the values was as mean ± SD (*n* = 3), *P* < 0.05.

As predicted via starBase, targeted binding sites was manifested in miR-888-5p and RB1CC1 (Figure 5(e)). Subsequently, descending luciferase activity of A549/DDP cells was clarified behind co-transfection of RB1CC1-WT and miR-888-5p mimic (Figure 5(f)). Moreover, repressive miR-888-5p elevated RB1CC1 (Figure 5(g)). Shortly, miR-888-5p negatively controlled RB1CC1.

### 3.6 Depressive RB1CC1 turns around the impacts of Circ-CUL2 elevation on A549/DDP cells

**Figure 6.**
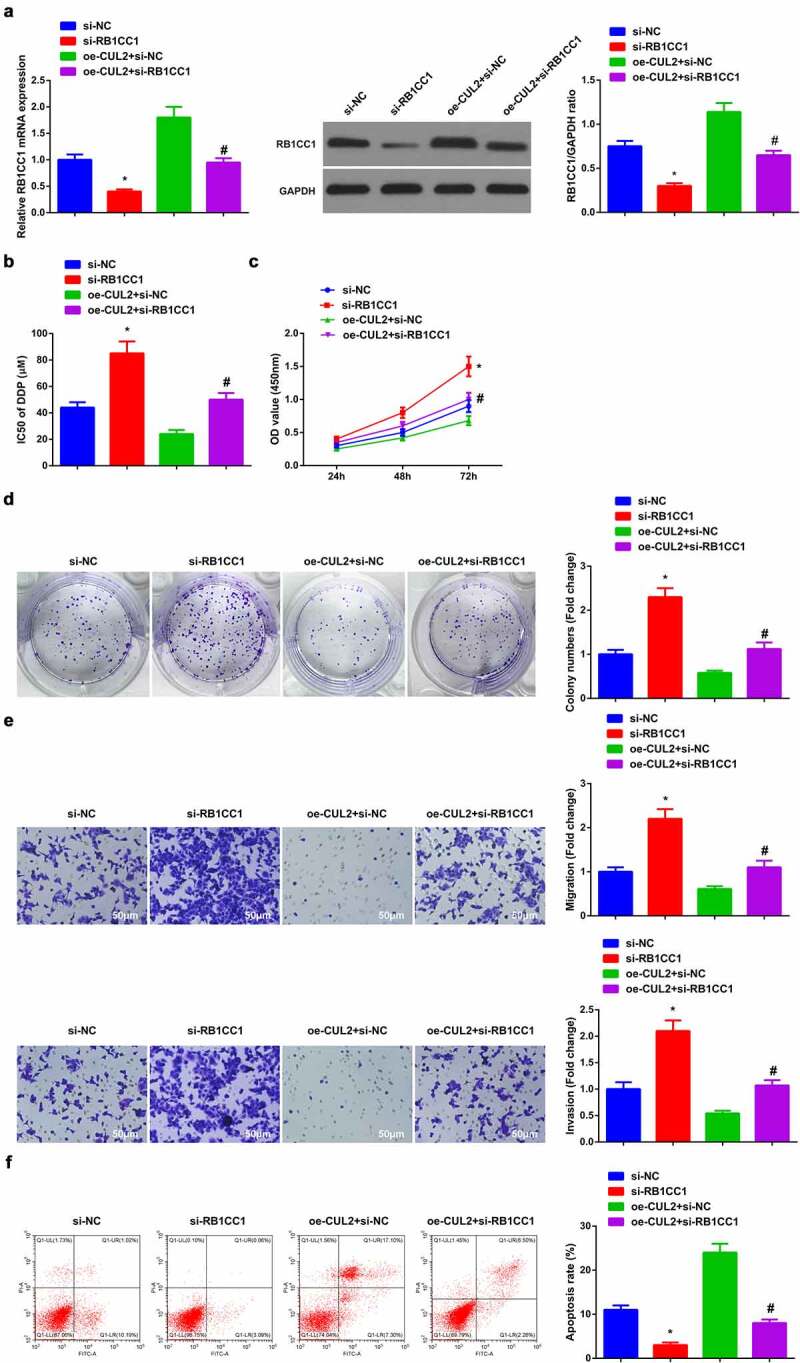
Refraining RB1CC1 turns around the impacts of elevated Circ-CUL2 on A549/DDP cells.

Next, exploration of the modulatory character of Circ-CUL2/miR-888-5p/RB1CC1 axis was clarified. Transfection of si-RB1CC1, oe-CUL2 + si-RB1CC1, si-NC and oe-CUL2 + si-NC was into A549/DDP cells, and the successful transfection was verified (Figure 6(a)). Meanwhile, depressive RB1CC1 turned around the repression of elevated Circ-CUL2 on IC50 value of DDP (Figure 6(b)). Moreover, behind descending RB1CC1, the refraining of Circ-CUL2 on A549/DDP cell advancement was of reversion (Figure 6(c–f)). Briefly, reduced RB1CC1 turned around the impacts of Circ-CUL2 enhancing on A549/DDP cells. In the meantime, it was also investigated the effect of circ-CUL2/miR-888-5p/RB1CC1 axis on DDP nonresistant cell lines, and the experimental results were similar to those of A549/DDP cells (attached Figure 2(a–d)). This further supported the conclusion.

(a) RT-qPCR and Western blot verification of successful transfection of RB1CC1 in A549/DDP cells. (b, c) CCK-8 method’s determination of the impacts of RB1CC1 on A549/DDP cell proliferation and IC50 value of DDP. (d) Plate cloning analysis of the impacts of RB1CC1 on cell colony. (e) Transwell analysis of the impacts of RB1CC1 on cell migration and invasion. (f) Flow cytometry analysis of the impacts of RB1CC1 on apoptosis. The data in the figure were all measurement data, and presentation of the values was as mean ± SD; * vs the si-NC, *P* < 0.05; # vs the oe-CUL2 + si-NC, *P* < 0.05.

## 4 Discussion

LC has been diagnosed as an extremely familiar malignant tumor worldwide, with surprising morbidity and mortality. In terms of LC, nearly 1.8 million newly diagnosed cases and 1.6 million deaths each year are present, and the deaths induced via LC takes up 19% of all cancer-linked deaths [22]. LC is regarded as the major reason of cancer-linked death, with a steadily enhancing incidence and has become a principal obstacle to human health [23]. Surprising recurrence and metastasis rates are considered main elements resulting in unpleasing prognosis of LC patients [24]. Despite the persistent perfection of diagnosis and treatment, the 5-year overall survival rate of LC patients is still less than 20% [25]. More importantly, drug resistance in LC therapy is extremely linked with abnormal oncogenes or anti-tumor genes, consisting of changes in biological features of malignant tumors, cell proliferation, metastasis etc [26]. Though DDP has been applied as an anticancer chemotherapy drug, all kinds of cancers (covering LC) may produce acquired resistance to DDP, which is a stumbling block to mitigating the efficacy of chemotherapy [27]. Moreover, the cytotoxicity of DDP remains a familiar side effect of DDP [28]. Hence, the study of the sensitivity mechanism of LC cells to DDP is vital for the combination of drugs on the grounds of specific molecular mechanisms to lighten the chemotherapy efficacy of LC.

DDP is a first-line chemotherapy drug in the clinical therapy of NSCLC [29], but its efficacy is seriously limited owing to DDP resistance in NSCLC cells. Hence, at present, researchers are concentrating on the latent mechanism of DDP resistance, for developing promising therapeutic drugs to rescue the chemotherapy sensitivity of NSCLC cells to DDP [30,31]. In this study, it was discovered that a brand-new circRNA, Circ CLU2, was reduced in NSCLC tissues and cells. Circ CLU2 was available to repress cell advancement via miR-888-5p/RB1CC1 axis with depressive DDP resistance in NSCLC.

CircRNAs and miRNAs refer to key tumor phenotypes, like proliferation, apoptosis and chemotherapy resistance, and CircRNAs act biologically via disparate molecular mechanisms. A familiar molecular mechanism is that circRNA controls cell phenotypes via spongy miRNAs [32–34]. In this study, detection of Circ-CUL2 was in NSCLC tissues and cells, clarifying the decline. Patients with elevated cirC-CUL2 manifested a better prognosis. Meanwhile, discussion of the impacts of Circ-CUL2 was on A549/DDP cell advancement with DDP resistance, affirming that Circ-CUL2 repressed cell advancement with enhancing sensitivity of A549/DDP cells to DDP.

MiRNAs are crucial in a great many biological processes, consisting of cell proliferation [35], and in LC advancement and metastasis [36]. In this study, a focus was on the downstream candidate miRNA of Circ-CUL2 (miR-888-5p), which has been manifested the elevation in hepatocellular carcinoma and acceleration of cancer cell metastasis [18]. But its character in LC has not been explored. Here, via experimental detection was discovered that reduced miR-888-5p restrained A549/DDP cell advancement with enhancing the sensitivity to DDP.

Moreover, a recent study has clarified that RB1CC1 is available to repress RCC cell development [21]. Similarly, RB1CC1 curbs prostate cancer [37]. Reduced RB1CC1 forecasts unpleasing prognosis of breast cancer [38]. In this study, it was discovered that RB1CC1 was the direct downstream target of miR-888-5p, and repressive RB1CC1 turned around the impacts of elevated Circ-CUL2 on A549/DDP cells. It was proved that Circ-CUL2 exerted its tumor suppressive influence via miR-888-5p/RB1CC1 axis.

## 5 Conclusion

Anti-tumor CircCUL2 is reduced in NSCLC tissues and cells. In the meantime, CircCUL2 represses DDP resistance of NSCLC via miR-888-5p/RB1CC1 axis. These results manifest that Circ-CUL2 is supposed to be a functional marker for NSCLC and a latent therapeutic target for NSCLC.
